# Supplementary material for: Role of miR-133/Dio3 Axis in the T3-Dependent Modulation of Cardiac mitoK-ATP Expression
Source: Int J Mol Sci. 2022 Jun 11;23(12):6549. doi: 10.3390/ijms23126549 (PMC9223604; doi:10.3390/ijms23126549)
Supplement: Supplementary file 1 [file ijms-23-06549-s001.zip › ijms-1766743-supplementary.pdf]

**Supplemantary Table S1.** Sequence of the oligonucleotides used in the transfection experiments

| Oligonucleotide | Sequence |
|---|---|
| miR-133a-mimic | 5'-UUUGGUCCCCUUCAACCAGCUGUU-3' |
| | 5'-CAGCUGGUUGAAGGGGACCUUAUU-3' |
| DS control mimic | 5'-UUCUCCGAACGUGUCACGUTT-3' |
| | 5'-ACGUGACACGUUCGGAGAATT-3' |
| miR-133a-decoy | 5'-CAGCUGGUUGAAGGGGACCAAAT-3' |
| SS control-decoy | 5'-CAGUAUUUGUGUAGUACAA-3' |

DS= double strand; SS= single strand. To increase stability, the RNA decoy nucleotides were modified by methylation in the 2' position of the ribose

**Supplementary Table S2.** Sequence of the primers used in the rtPCR experiments

| Gene | Sequence | Accession number |
|---|---|---|
| MitoK | F 5'-GGAGCCTAATAAGCTTAGTCTCTTCA-3' | NM_001014098.1 |
| | R 5'-CGAAAGGTTCTCCATACCAGTC-3' | |
| MitoSur | F 5'-CTCAAACATCGCTTTCAACTGT-3' | NM_001007796.1 |
| | R 5'-ACATGAGGTCTCCCCCTTTC-3' | |
| Dio3 | F 5'-TTTTGGTACTTTTTGGCTTGC-3' | NM_017210.4 |
| | R 5'-CTTGTGCGTAGTCGAGGATG-3' | |
| Hprt | F 5'-CCCAGCGTCGTGATTAGTGATG-3' | NM_012583.2 |
| | R 5'- ACTCTTCATTCAGGCCCTTG-3' | |
| Hmbs | F 5' TCAGSTGGCTCAGATAGCATGCA-3' | NM_013168.2 |
| | R 5' TGGACCATCTTCTTGCTGAACA-3' | |
| **miRNA** | **Sequennce** | **Accession number** |
| 133a-3p | 5'-TTTGGTCCCCTTCAACAAGCTG-3' | MIMAT0000839 |
| SnRNA-U1 | 5'-CGACTGCATAATTTGTGGTAG-3' | |
| SnRNA-U6 | 5'-CGCAAGGATGACACGCAAATT-3' | |

## **Figure S1. Rat mitoK promoter region:** in red THRa1 consensus sequence

>NC_051343.1:109717595-109722595 Rattus norvegicus strain BN/NHsdMcwi chromosome 8, mRatBN7.2, whole genome shotgun sequence.

-5000 TGCATCCTTCAGGAATGAGGGCCTCAAAACAATGCCCTTCAGTCCCAACAACCCAGAGAGTTGTCAGGCTTTTTGCAAAACAATGCCAAACTAAACTGTCAGT
CCAGCACCCCTTACCTGACTTGGGAGACTGACGGAATGGTTAGTTTATTTGTTCGTTCGTTGCTCTGAAGCTTTGTCCTTAATTCATTCATTTCTGCATCTTTAA
ACTGGAGTTCAGACTGCAGTGATTGGAGCTGAAAGCCAAAGATATTTATGGTAACACTACCATACACACACACAAAAATACGTTCGTGGTGGTGCACACCTT
TAATCTAACACTCGGGAAGCAAAGGCAGGGGTGGGGAATCTCTGTGATTCCACATATTGACTTCTGAGGCTGCCAGGGTTACAGAGAGTCTGTCTCAAAAA
CAACAACAACAATAATACATTCCTTAGCTTCCAAACTTCAAGTCAGTAAAGGAAAACAATGAAAATACATTATCAAGGTCTGCTGTAATATAAATGTCCTCAA
ATATTAGCCTGTTTTTCTTGAAAAATACACTAGAAAGATGTAACAGACTCTATTGTGGAAGTAATCTATGAGGGCTCAAATACCAGCCCATTGCCTGTTTACCT
TCACTGAGTCTAACAGGAACTTAGCATTCTCGGCTGGGACTGCGCATTAGAGAAAGTGATGGCTGAACAGTACCCAGCACAGAGCTGGGGAGAGCGATGTC
TTATCTCGGCAGTAACATGCCCAACCCCACCCCACACTCTGCTCCCCAAACTGCTGCCGAACTCTCAATCCTCTTCAGCTTTCACAGGGATTGGCTGCTAACAT
CATCTGTTAGTTCCTTAGTGGTACCGGGGTCTTCTTGGAAGTCTGAGCAGATTATCTGGAAGTGTTACCTCAGTCTGTGTGTGAGTATATGCTGAGATTGGCC
TGTCTGTGTGTCAGGAAACTAGAAAGAGGGTCAGAAGAGGGACTGAAATCTTAAGCAAGTGAGGAAACAGAGTAAGGGATACATGTACTAGGAAAGCAA
AGGGAGAAAAAGCAACTGTAGCTCAGGTGGAAGGGTTTACCTGGGATGCACGCCCAGGGCTGAGTAAGGAGGCCTGGCAGCGGTTATGCCTGCAATCCAG
GGCTCAGGGGGCAGAAACAGGAGGTTCAGAAGTTTAAGGACATCCTTGGCTACAAAGTGACTTTAAGGCCAGCCTGGGCTACATTACCAGATCACAAAAAA
ATAATGTTTGGGCTTCCCAGAACCTTCCCTCTGGTGGAGCAAGGGTATGTGCACAAAGAACTTGCTATGGGAACAGGTAATGGAGGCGGGCACTCAAGCTC
CTGTCCCCGCCACTGCTCCCAACACTGGAAAGGGGGCGGAGTGCACATCCAACTGAAGTATCCACGGGTTCTAAAGGAAAGTGGTGCTGAGACTGGTACCT
ACCATTTCTCTTTGAACACGAGCTCCCTTTCCTCCCTCTGCCTGTGACTTAAATGAGACCTCTTTCAGAACCCGGCTGAAAATGGGCTGCCTCTTTAAAAATTTT
AAGTTTGAGATGAGGGAGCCTTTCATTTGGCTTTCTGAAAAAGGATCTCACTGTATAGCCCAGGCTGGCCTTGGACCCATGGTAACCCTGTTGTACCAGCCTC
CCAATTTATGCCCAGCTGAGGAAGCCCAAATCAAAGCTCTGGAGTTTTGAAATGTATGAGTTCATCCAGTCAGAACCCCTGCTGATAGAGCAACAGGTCTCA
CAACAGAGAGCCTGAGATGTCTGGTTTACACCATCTGAAGCCCTACTATGCCCCTGTTTTCCTAGACTTGGATGTGATAGCTAATGTATTTCCTTCTGGCTAAG
CCGAGCCAACTGGATTTCTAACCTACCAAGGAGCCTAATTCAAGCCTATCCTAGAGATTCTAAGCTAGTGACTTGTTCCTGCCAAGAACCACAACCACAGCAA
GGCAGGGCCTCATCTTACTCCTTGTCCAAAACAAATGCAGACCACGGAAGAAAGGCACATACCACCCACTAAGAGTGGAGAGCACTTAGACAATGGCTGTG
TATTTCCAGGCAAGGCTGGTTGGTTACTGCTTTTATTTTGTTTATTTTTTAGACACAGGGTCTCACTTGACTGGACTGGAATTCACCCTGCAGTGAGCTGGCTC
AGAGCTCACAGAGCTCCACCTGCCTCTGCCTCCTGAAATTAGAGGCATGTGCCAGCTTGCCTAGGCATAGTATGGCTTCTACTGTAACTCAGCGGTAGGCAA
CACTACTGTCCGTCGCACGCAACACAAATGTTCTAGCTCACCTTTTTGGAGAATTCCTTTTCCTTTTCACTGAGTGCTTGAGTCTTTTCTTGCTCAAGAAGAAAA
TGTGATCTTTTCTGTTCTTCTAGAACGGATTCTGTCTGATGCAGTGAGTCACGCAAAATTTTAATTTCTCCATTCTTAATTAGGATTTCTTCTTCCATTGCTTTCA
GCTGCAAATTAGGCCCAATTAGCAGATATTCAGCAACAACAGTTTCCGAATACGTTCACCATTGACATAGATTTTTTCAGCTTTAACACCACAGATCACACCTT
ACATGGACTAAAATTCCATGTTATATTTCTGGGTTTCTTGGGCACCTACAGATGACCCACATGAGAGATGTATGAGAGCTTGCAACACTAACAAAATAGCAGC
CATTATGATGGCATGTTCCTCAGATAAAGACAGGCTACAGGCTGTGGAGGCTGCAGAGATGGCTTAGCAGCTAAACGCACTCACTGCTCTTACAGAGGACCC
AGGTTCCCTCCCAGTACCCATGTCAGGTGGTTCACACCAGTTGTGACGATGGTTCCAGAGGATCCAACACCCATGACATTCACAAGTACCTGCATACATGCAG
TGCATATAAACTCGCACAGGCTCACGTATGTACACAGAGTAAATAACGTAGCAGGCATTCCTTCCCCACCTCCTGTCATTTCAGAGAGGTGACTCTAGGTGCT
TGTTTTAGATATCTTTATGTATTTTACCCATGTTTTCTTAATACATAAACACATGAATCAGCTACAAATACAACAAATTAGGGCTGGAAGTATGACTTAACTGT
AGCTATGTATGTGTATAGCATGTGTGAAGGTAAAGTCCTAGGTTCAATTCTCAGTATTAGAACCAAACAGGCAAAAAAGGAAACCCAAAAGTTACACACCTT
TTCTTTAAGTTCTCTGTACTGTATCTGAAGTACTTCTAGTTCAAAGTTATCTTTAACTGGAATATCTTCTCTGTTTTTCCTTATAGGACTATTTGGTAACCCATCT
AGTCGACGGACCTTATGAGCACCTGTATAAAAAAATAGTCCCCATTAGGTTGAGCATGGTGGCATGTAACTGTAACCCCAGCACTTAGATGGCTAAGGCACC
AGGATCTCAAGTTCAAGGCTAGCTTAGTTATAGATGGAGAACCTGTCTGGAAGAAAATACCATGTATATATACCTGGGGGCTCACTATATACCCACCATTGTC
ATAAAATGCAAAAGGAGTCCAAGTTGAGATAGACCCAGTAAACAGTATTTATTAGTTCCGATCTCAGAGCATGATATAGAGAATCCTTCTGGCATAGCATAT
CTGAATATTCCCCCTTTCGATCCCCACCCACTTTCTGTAGTCT

-1359 C**AGCTCATGGCTG**AAAGGAGTCTGCATTGGCTGTGCCTCTATTATGAATACTATTTACTGAACACACTAG -1289

GCACAGGATCTGCTGGTTTTTAAAGACCGATGGTGAACAAAATGGATACGTCCCCAGGGTGGGGACACTCCAGGGTTAGAGTATGGGATGATGAGTGACG
TAGGGCCAAGAGGTGGGGATGGGGAGGTGCTGCCTTGTACCACTTCGTGGAAACTAGTGCCTAACCACTGTGGCATAATACAGCTGGTCAGCAATGAGTG
TTTCCCAGGCAATATTGCTGGAGAGATGGAGGACCGACCACATTTCCAGGGATAATCCTATTTTTCGGATGCTTTAAATGCTAAGTACATTTGCAAGATCTCC
ATCAAACCCTTCTGACACTTAACTTACTCCCCAAGAGTGTAAGCTCTAAGAGAGAAAGCAGCTTTGGCATCGCTCGCCCATTGCTTATGGCTGTTGCCATTCC
TCACCTTTGGGCAACCAGCATTGGTGGTGTATGAATCTCTCATCTCTTGCCTCTGACAGAAGCTTTCTGCCCAAGGGTTTCTAACAATGGGGTGCATAACATT
TAAATCCTCTCACATGCGTTGTGACGGTCCTGAGGTGATCGTAGTCATGTTTCAAGGAAAATAATTAATAGTCTAGCTGTCTATATTTCAAATCTGCATATGT
GAGGCCCTTTGACTTTTTTTTGTCTTACCACCAATTTAGAGGGTTGTTTGAAACAGAACCGCGTTTCGATCGGGTGCTATTTCGTTTTTAAAAGTGAGAGGGT
AACGTCGGTGCAAGCAGCCAGTACCAAAGCTACCAAGCTGAAGAGGTCTGCACCACCCGCCGTGGGAACACCCCCGCCCCCCCCCCCCCCCGGCACTCAC
AGGACAGGTTCGGAGGCGCGACCGGACACTGGCTCAGGGCCTGTGACGCGAGGATGTCCAGTTCCTCTAGGTCGTCCGCAGTAAATTCCGCGTGCTCGCC
GAATGGGTCTTCGGGGTCCGGAACTGTGGTCTCAGAGAAGCTCCGGGCCCGCTTGCTCGGAGGGTTCTCGATGGACCGAGACAGACCGAGGAAAGGCTCC
AGGCCGCCACTCTGCTTCCTGTGGCTGTTCGGTGCGGGAGTCCCGGCCATGACTACCTTCAGCTCGCAGCATCTGAGGACGCCAAGCACAGGTTGATCGGC
AGAAAGCCTGAGATGGCCCAACCGCCCGCGCGCCGCCGTTCCCCTCAGCTACTAGCGTCTCCCCAGCCTGCGGGCCAATCAGCTGCCAGCTACCGGGCACA
GATGGGTGACGTATACAGAGCGCGCCGTTACCCGGGACAGGTTCCTGCCTTGCTGGGCTCAGTACCCTGAGGT

**Figure S2. Rat mitoSur (ABCB8) promoter region:** in red THRa1 consensus sequence

>NC_051339.1:c10788598-10783598 Rattus norvegicus strain BN/NHsdMcwi chromosome 4, mRatBN7.2, whole genome shotgun sequence

**-5000** CTTCCTGCCCAGAGGCTTGGGTGGCCCTCCCCTCACC**CTGGCCTGACCT**TCCTCGACCAATCAGGATGCC **-4930**
TCCTGATCTAGAAAGCCCAGTGACAGCCCTTCCTATAAACAGCCATTGTTTTTACCACCCGCATGTGACTGTAAGTCACTGTGTGACCCATCGTGTGAAGCTTCC
TTCACATGGGTCAGAGCCAAGCTTACTAAGTGCCTGAACCTGCCTCCTGACACTACTCCAGGCCCAGGGCAGCTTCCGTCTCCCTGCTCTTCTCAGTTGGTCCTT
CCATGGCCCCACTCTGTCCTGTCCTGTACAGCATGCTTACTGGGGGGATATGCAGGGCCTCCCTGTAAAACACCTGGATGTCCCAGTAGCTGAAGAGATTGCA
GACCGAGCGCAGCAGCTGAAACAGCCAGAAGCCAGCAGCCAAGACTAGGAGGAAGACCAACAGAGGACTGCCATGGATCCTGCAAGAGGTTGGAAGAGAG
GGTAACTTAAAGATGGGAAACCCACAGAAAGGAAATGGTGGCAAGACCCTCCAGTCAAAGAGGGGCTAAGGAGGGGGCCCTCAGTGAGCCCTTCAGAAGTG
CCCACCCAGTCAACACTTCCCAAACAACACTCATGCCCTCTGCTATTTGTGTTGTCAGTCTCCCACAGCTGTACAGCGGAGTTTTCCAGAAGCTACAAGACAAGC
AATGACGTCACATCATCCTGACAGCCCATGCAGTGTGCACTTGTGCATTCCTAAGTTTTAGAGTTGTCCCCAAGGCTAGGAATGTGGCTGAGTAGTGCAGCACT
TACCCAGCATGCCCGAGGCCCTAGGCTTGCTCCCCAGCACCAAAAGTAAACAGTTGTCCTCACCATTATTAATTTCTAATAAGGTAAACAGAAATGTGACCCAT
AATTCAAGGCTCTTTGAGTTTCCCAGTTGACTTGGGGTATGTTTTTAAACACTGGGAGTTGATCTAGACCAAGGGGTAGTGTGTGGAGGTCCCTTTCTTCCTATC
CTTTGTGAGCTTACCCCCCCCCCCCCCCGAGGCAGCCACTAGACTGTCACTTACTTCTCTGCACACTGGGCTGAGGGTAGAATGGCATCTGACAAGGTCACTTT
GCTGTGCAATGGCCCAGGCCTTGTATGGTTCTTCGGTTGGTTGTTGAAGAGAACATTGTAATCCACGCAGCGAAGGAGGAAGGTTGTAAAGGTGACAATGAA
AATGAACTGTCTGAGGAGGGGATGCAGAAGAGGTCTGGTCAGGCTGAAGGCATGGCAGACACCTAGCCTAGTCACAAGACAAGCTGCAGCTCTCTATGGCCC
CCTGACCCTTCAGTGACCCAAGCCTAGAACTGAAAAGGAGGGCTGTGGCAGAACTCAGCCCAGATATGAAGCCCCAGATTTCATCTCCAGCACCACACAAACT
GGGCACAGTAGTGTGCCTGTAATTCTAGTGCATAGGAGTTAGAGGCAGGAGGAATTCAAGCCTAGCCTAGGATACATGAGACACTGGGTCAAAAATAAATAA
ATAGAGCGAACACCGAAAACAAGAAGAGAGCAGAGCTTAGGGCCAGGACTGGCTTCTCACCCCAGCTGGAAGACATCCTCCAGCAGGATACAGGCAAAACC
GTTCCGCTGGTGATAGCTGTAGATGTGGCAAGTTAGTCAAGGAACATGCCTAAGCCAATGCCAATGGACCCTTCCTATAGAAACTTCTCCCACTCTCCCCTGCT
CCACAATCCCCCACCATGTCCCTCTGCATACTGGGCACAAAGGATATCTTGGTGAAGAAACTGTCCAGGTTCTGGATGTGATGCCAGGAGCCTGGGCACAGAG
AAACAGTGGCAGGACTTGGCATTGTCCCATTTGTGATTAAGTTCTCTATCAAACCCAAACCTGCCCGGACCCCCCAAGCCCCTCCAACCTTCCGTACACATACAC
TCACCACGGAGCCCTTCAGGTACATGGAGCAAAGGTTGGTGGTCCTCCCCATGGACGGGCGAGGACGAATCTTGAGACCCCTCAGGGTCACAGTCCTCTAGCC
TTTCATAATCCTGCTCAGGGATCAGAGGGCCTATCCGCAGCCCAGGATGGTCCTGGGGGCACCGGCAGGAGGGAGGGGGAGTGACCGAGGCCAGGGGTGG
GGTGGAGTGGGACCCCCATGAGGGGGAAGCAGAGGTGTGTGTCATTGTGGGAAGGGCCTGTGTTGGGGGTGTGGCTGGGGTGGGTAAGGCATTGAATGGA
GGCTGAGAAGCCCCTGCCTCCTGAATCACCAGGGAGGGGGACCCTGAGGTGGGAGGGAACAATGAAGAGGAGCAGCTTCTTGTACGAGGGGCAGGGGACA
GAGAGAAGACAGAGATCCTCCCTCCCCCAAGTCCCCGACACGGAGAAGCAGGCAGTGGCAGTGCCATAGGGAGCAGAGGCACTGAGCTGGGCCCCAAGTCT
CCCCACCGCCCCCGCTGCCTCCTGCTCCCCCCCACCCCATTCGTCTCACCATCAGGCGGCGGCTTCTCTTGAGACGTCGAAGGGACTGCTCTGCTGTTGTTTCCT
CAGAGGTCTGTGAAGCTGAACAGGGACTACAACAAGGACAACAACAGTACCTGGTGACTCAGGCTGGGCAGGCCTTAGGCATGCCAGCCCTCAGCCACCTGT
GTGATCACTCACACTCCTTGCAGAGCAACCTGGGGCCCAAGCTCCGTGCCTGGGAGGAAGGCCGGTTTGGGAGTCAGGGGTTCATGAGTTATTGGACTAGAA
GAGACACCTCTGGTCTCTGCTTTCAGTCCTCAAGGGCATGAGTGGTAAAGACAGGGCAGCCGCAACAAAAATAACAAGCGCAGAGAACAAAATCCTATCAGA
GGGTTCTAGAGTCTGGGGTGGGGGTGGGGAGTAGTAAACCTACAGTTACATCCCCTTCCCTTCACCCTGACCGGCACTGTTCAATAGAGAATGAGTTAAGACC
ACCTGCCCAGAAAGGTGATCCATTCAAAATTCCCAGCACTTAGGAGGAGATGGACAGCAGGATTAGGGGTCTGAAGTCATTTTGGGCAACATCTGACCCTACC
TTTTTTTCATATTTTCATATTTTCTCATATTTCATTTCTCTGAATGAAATAGGGGAAACTTTGCGGTCAGCTAGGGGAAAACAAGAAGGAGTAAAAGGAGGGAG
TGCTAACCATCTTGGATTTGAGAAATTATTTGAGGAGCTTAAGTAGAAAAGCTTGGTTCCTTGAGAGTTCATTTATGTCTTCTTCATCCTCGTGAACCCCAATAT
ACTCTCCTCCAAGTCCTGACCTGGTGTTGGGGCCTCCTCAAAAGTGTCAGCAAAGGAAGGTAGGAAGAGGTGAGAGCCTTGGAAGGGTAATTTGGGTATGCA
AGGATGGACTGGCTCAGTTGAGTTCTTTGTTGTTGCCTCATCCAGGAGGAGGAGGCCCAGGTGGCTGGTCTGATGGCTTGCCCAACGCCATTCCCAAGGTCCT
GAGTCACCAAGTGCCCTACTGAAGCCCATGCCTGAGATGCTGACTTAGCAAGCAGGTGTCAGGGCCTTCAGCATTCAAGCCAGTATCTCAGAAACAGAGAGG
AAAACAGTGCTTCCTGGAGGAACGTGGAGGACATCCATGGAACGTCCCACAAGTTTAGCCCAGACTGCCTGATTTTCTCATATAACCAGAGCTCATGGCTTCCA
TACCTTTGTGGGTAAGGTTCAAAGCTATGCTTTGTGGCCAAATGGAATCCCAAAGAAGTAAAGGGGTGGGGGGTGGGGGAGCAGAAAATTCCCATGATCTTT
CTGAGACCTCTAGAGAGATAGGTTAAATGTGGCTCTAACTGGTACCTCCCTCTGACATTTCTTACACGGCATTCTTCATAGTATATGCTTGTTTAAGGCTACCTCT
ATACACACCCTGATCTCAGCCCCAATCTCAAACAAATTCTGGCTCATTGCTCTGTCTAGTTTCACTCCACATTCTCATATCCAGAGCATCCCTTTGCGACCCTCGG
CTTAAGGAGAATTGGATCCCGGAAGCACCACCGATGACAACGATACTAGTAACACTAATAACAATCAATGTTCAAAAGTCCAGATGGATTACCAGAGAGCAGA
TGCGAGTTAGAAGCAGCGCCCCCTGGTGGTCAGTGTTCAATAGCGTGTGTCTTTCCCTGACGGACGCGTGGAGGGAAGCAGGTATGAGTCAGAGCCACTCCC
ACCAAGAAGCCACAGCCCAGTGGAGGGGGCAGTTAAGAAAAATAATAATTACACAGAAGGCATAGTGACTCAAGTCTGTAATCCCAGCACTGGGAAGGAAG
GATGGAGAGTTCAAGACAAGCCTGAGCTATAAAGAGTCTCAGGCTAGTCAGAGATAATGTAGCAAGTCTCCGGGGTACTTTGGGAGATAATTATAGCAAGAA
TAAGCTTAGCCCAACTTAGCTATACGCTTTTGAGCTGGAACTTTAGGCCAGTGATTCAGATTCAAATGAGGAAGGAACCTATAGGTTTGATCGTAGGTTGTGCA
TTGATAAACTTCATGACCTACATACTAATAATTCCCGCTAACTAAGTGTCAGTAGCAGAATCACACAGAGAATTTTCAATGCATGTGAGTGGTGGCACGTCACG
GGCATGTATGTTTTTGTTTGGTTTGGGTTGAGAGTGTGTGTGTGTGTGTGTGTGTGTGTGTGTGTGTGTGTGTGTGTGTGTGTGTGTGTGTTGCTTTTTTAACTC
CCAGGTCTGTTTTCACTCATTCCTTCTCACGGAACCACCACTCTACACTCCCTGAAGATGCCAGGGCACATAGAGATTGTCCATAATAACTCTAGAGAAACTGCT
CAAGGTCAGAAACGCCAGGACATCCTCGAATGTGAGCGTGAACGACACACAACCTCTGCCGGTCACCCGACCGTAGGCCACTGAGCAAATGGTTGCCAGAGC
TGTTTCTGCTCGGTCCCGCCTACAGCCAATACCTTATCCAATCACAGTTTGTGATAAGACTGATGTGTCTTTCCTCCAATCACCGCGGA **0**
